# 

**Published:** 1801-05

**Authors:** 


### TO CORRESPONDENTS.

*We have received many useful and friendly hints from some of our Correspondents, but are prevented from making the use we wish of them, on account of their being anonymous. The only occasion, as we think, on which a Gentleman can find it necessary to write anonymously, is when he is desirous of communicating unsuccessful or dangerous practice; and even in such cases, we also think, a private note should be addressed to one of the Editors, containing the real name. We assure* " A Reader and Contributor," *that we never will, knowingly, admit the Communications of any* " common advertifing Quack;" *and if it has ever been done, it was inadvertently. If this Gentleman had favoured us with his name, we could have explained some things in his letter to his satisfaction.*

*Mr. Bailey's Communication with the Engraving, shall appear in our next.*

---

### *Advertisement to the Medical World.*

THE Proprietors of the periodical work, entitled THE LONDON MEDICAL REVIEW and MAGAZINE, having determined in future to confine the object of that work *solely* to a full and comprehenfive analyfis of NEW MEDICAL BOOKS, domeftic and foreign; and having agreed to recommend their friendly Correfpondents to tranfmit their future communications to the MEDICAL and PHYSICAL JOURNAL, the Editors of the latter work announce this new and improved arrangement with much fatisfaction, and earneftly invite the Correfpondents of the London Medical Review and Magazine to honour them with their communications; affuring them, that they will on all occafions be treated with due refpect and diftinction.

The

The Editors of the MEDICAL and PHYSICAL JOURNAL take this opportunity to repeat their acknowledgements to the medical world, for the continued and increafed fupport with which their exertions have been attended; and as THE MEDICAL and PHYSICAL JOURNAL will continue to be carried on *precisely upon the same plan, and with the same spirit as heretofore,* the Editors venture to promife their readers a confiderable addition to the value of this work, from the acquifition of the correfpondence which has hitherto been peculiar to the LONDON MEDICAL REVIEW and MAGAZINE.

This laft mentioned work will in future be publifhed as ufual, on the firft day of every month, under the title of the LONDON MEDICAL REVIEW, and will recommend itfelf to a continuance of the patronage it has been honoured with, by the copioufnefs and the independence of its criticifms on every new publication which is connected with the ftudies or purfuits of medical men. The plan of this work being, therefore, wholly different from that of the Medical and Phyfical Journal, the profeffional reader will be able to indulge his peculiar views in the purchafe of a periodical medical work, at half the previous expence. Thofe who are in queft of new facts, obfervations, experiments, and cafes, will confult the MEDICAL and PHYSICAL JOURNAL, in which they will alfo meet with a Retrofpect neceffarily brief, of all the novelties in medical literature; and thofe who would enter more at large into the critical difcuffion and hiftory of the practice of medicine, and its collateral fciences, will probably feel a ftrong intereft in the perufal of the LONDON MEDICAL REVIEW. Many gentlemen, who, not regarding the expence, determine to gratify themfelves by reading both works, will have the advantage, in confequence of this arrangement, of not purchafing the fame intelligence twice over. In every point of view, the Editors of both works confider the arrangement they have adopted as matter of congratulation to their friends and correfpondents, and they hope the public at large will be of the fame opinion.

*London, April* 25, 1801.

